# Innovations in the Design and Application of Stimuli-Responsive Restorative Dental Polymers

**DOI:** 10.3390/polym15163346

**Published:** 2023-08-09

**Authors:** Ana Paula P. Fugolin, Bao Huynh, Sivashankari P. Rajasekaran

**Affiliations:** Department of Oral Rehabilitation and Biosciences, School of Dentistry, Oregon Health & Science University, Portland, OR 97201, USA; huynhb@ohsu.edu (B.H.); purushot@ohsu.edu (S.P.R.)

**Keywords:** resin composites, restorative materials, stimuli-responsive, bio-responsive systems, intelligent dental biomaterials

## Abstract

The field of dental materials is undergoing rapid advancements in the pursuit of an innovative generation of dental polymeric restorative materials. There is a growing interest in the development of a distinct category of dental polymers that transcend the conventional role of inertly filling prepared cavities. Instead, these materials possess the capacity to actively detect and respond to alterations within the host environment by undergoing dynamic and controlled molecular changes. Despite the well-established status of stimuli-responsive polymeric systems in other fields, their implementation in dentistry is still in its nascent stages, presenting a multitude of promising opportunities for advancement. These systems revolve around the fundamental concept of harnessing distinctive stimuli inherent in the oral environment to trigger precise, targeted, predictable, and demand-driven responses through molecular modifications within the polymeric network. This review aims to provide a comprehensive overview of the diverse categories of stimuli-responsive polymers, accentuating the critical aspects that must be considered during their design and development phases. Furthermore, it evaluates their current application in the dental field while exploring potential alternatives for future advancements.

## 1. Introduction

Groundbreaking advances in dental restorative polymeric materials have been made over the last seventy years. These advancements have paved the way for the development of the minimally invasive operative dentistry principles that are currently recognized. Initially, the focus was on improving the mechanical properties, reactivity, chemical stability, handling characteristics, biological compatibility, and polymerization stress of dental polymers. Through dedicated efforts, benchmarks were successfully reached, leading to the translation of several technologies into clinical practice. Despite these achievements, restorative procedures still face higher failure rates than desired. This is primarily due to the complex nature of the oral cavity, which represents one of the harshest microenvironments. The oral cavity harbors more than 700 types of microorganisms, encompasses diverse living tissues, experiences frequent temperature and pH oscillations, contains enzymes, proteins, immunoglobulins, and a wide variety of electrolytes. In other words, dental polymers and dental tissues are subject to continuous physicochemical and biological challenges that compromise the longevity of the restorative procedures. However, the complexity of the oral environment also offers abundant stimuli that can be utilized to initiate organized and systematic actions in responsive systems that, ultimately, may lead to enhanced clinical lifespan of the restorative procedures and preservation of the living tissues [1].

Stimuli-responsive polymers possess the inherent capability of sensing alterations in the environmental conditions, effectively processing the sensed information, and subsequently initiating ordered and orchestrated actions [2,3]. These systems have been extensively studied over the last twenty years for a diverse range of applications, such as biosensing, smart coating, artificial muscles, tissue regeneration, and on-demand drug delivery [3]. In the field of dentistry, there is a growing interest in the development of resin composites that not only fill the cavity and exhibit the capability to withstand the harsh oral environment but also can actively interact with it. This represents a promising strategy to effectively extend the clinical longevity of dental restorations.

The field of stimuli-responsive dental restorative materials remains at an early stage of development and is rife with opportunities. The challenges encompassed in the development of stimuli-responsive dental materials are mainly related to the inherent characteristics of polymeric dental networks, which are primarily engineered to optimize mechanical strength and, consequently, lack the ability for dynamic reprocessing of chemical bonds. In order to withstand the challenging conditions of the oral environment, dental polymers possess a densely crosslinked polymeric network, resulting in an elevated glass transition temperature (Tg). This characteristic combination enables the development of a material that exhibits impressive resistance to the thermomechanical challenges encountered in the oral cavity. However, it also restricts the mobility of polymeric segments/chains, consequently demanding a significant amount of energy input to induce any form of displacement or conformational change [2].

To undergo nanoscale changes, such as molecular bond rearrangements, cleavage, motion, and morphology transitions, a delicate interplay exists between spatial characteristics and the energy necessary for these transformations [2]. The extent of spatial restrictions directly influences the magnitude of energy required for a polymeric network to transition from one state to another. Consequently, the generation of an adequate energy input requires the application of extreme stimuli, which frequently prove incompatible with physiological conditions. Furthermore, these transitions in the molecular architecture must take place without compromising the mechanical integrity and functionality of the polymeric networks, even temporarily. Additionally, it is a formidable task to ensure that the responsive moieties are readily accessible to interact with the target stimulus, rather than being buried within the dense and brittle polymeric network. These requirements pose a significant challenge in the development of responsive dental polymers, regardless of the stimulus that they are responsive to.

Nevertheless, in spite of these challenges, there exist elegant chemical approaches that, when effectively employed and associated, can overcome these obstacles and pave the way for the advancement of the next generation of dental restorative materials. The objective of this literature review is to present a comprehensive overview of physical, chemical, and biological stimuli-responsive polymers, highlighting key considerations for their design and development processes. Additionally, it evaluates their current applications in the dental field while exploring potential alternatives for future advancements. The searches encompassed the databases Scopus, PubMed, and Google Scholar, focusing on articles written in English and published within the last ten years. The keywords used were “responsive dental materials”, “smart dental polymers”, and “bioresponsive dental biomaterials”. Supplementary articles were identified by complementing the database searches with a manual review of the reference lists.

## 2. Stimuli-Responsive Polymers—Properties and Classification

**Figure 1:**
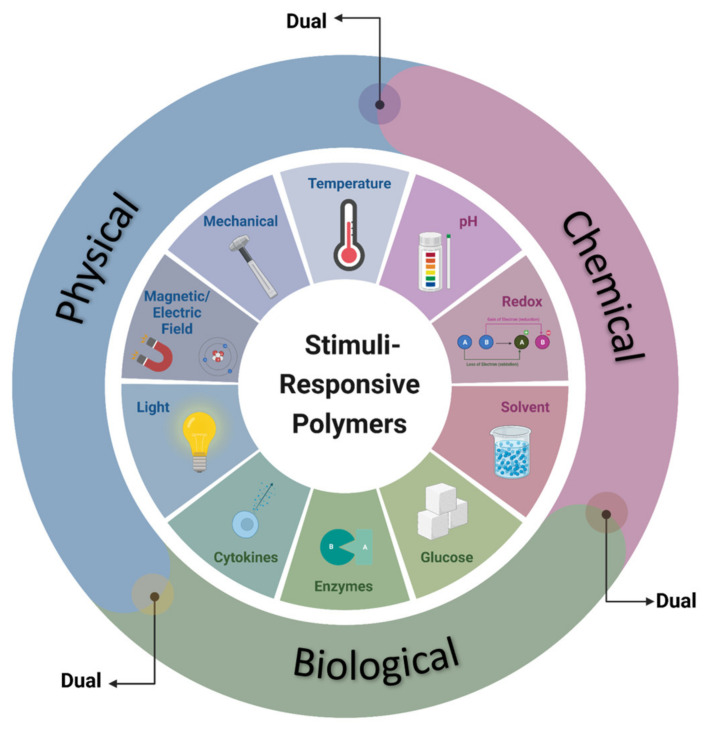
Illustration depicting the various categories of stimuli-responsive polymers based on the nature of the triggering stimulus.

There exist diverse categories of responsive polymeric systems that can be classified based on the stimuli that trigger molecular transitions in their networks. These stimuli may encompass physical, chemical, or biological factors. Furthermore, these materials can be categorized as single-responsive, dual-responsive, or multi-responsive, depending on their ability to respond to one, two, or multiple categories of stimuli, respectively (Figure 1).

### 2.1. Physical-Stimuli-Responsive Polymers

Polymers responses to physical stimuli are essentially based on modifications of the polymeric chain dynamics and molecular interactions promoted by mechanical stress, changes in temperature, light exposure, and induction of magnetic and electric fields [1,4].

#### 2.1.1. Mechanical

Mechanical-responsive polymers utilize mechanical energy to induce chemical transformations and bestow the networks with self-healing capabilities [5]. In general, elongation and compression are the most common forces that the polymers are subjected to and, consequently, used to trigger conformational and chain alignment changes, disruption of supramolecular interactions, or formation/disruption of excimers [5]. The mechanoresponsive materials are based on scissile or non-scissile transformation [5]. In scissile systems, covalent bond breaking, including selective cycloconversions and ring opening reactions, is triggered once the polymeric network is mechanically stimulated [5]. On the other hand, the non-scissile systems rely on conformational changes, (de)construction of aggregates, and breaking/reforming of non-covalent bonds [5]. The most recent advances in mechanical-responsive polymers have focused on not only restoring the network integrity but also enabling the self-healing systems with capabilities of tracing the damage extension and elapsed time by the controlled release of small molecules with mechanochromism or mechanoluminescence properties [5].

**Figure 2:**
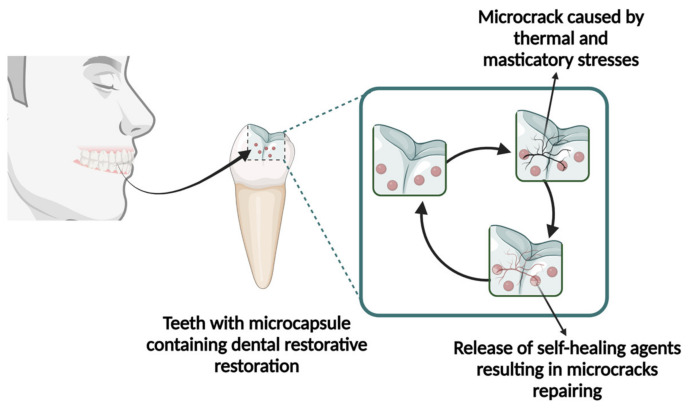
Schematic representation of the microcapsule-based self-healing dental composite mechanism. Essentially, the microcracks generated by thermomechanical stresses are responsible for triggering the release of the healing agent, which flows through the cracked area and seals it.

In the field of dentistry, the advancement of self-healing systems for the repair of microcracks caused by masticatory forces and thermal stresses is currently in its early stages. Among the different approaches explored thus far, the extrinsic strategy has received the most attention (Figure 2). This method entails sequestering healing agents in microcapsules, which are then incorporated into the polymeric network. As the microcracks are formed, the microcapsules are ruptured, and the healing agent flows through the damaged region, fills it in, and polymerizes [6]. In general, microcapsule-based self-healing systems utilized in dentistry are based on poly (urea-formaldehyde) shells encapsulating triethylene glycol dimethacrylate (TEGDMA) [7,8]. The toughness recovery achieved through this approach ranges from 70% to 90% [7,8,9]. The constraints associated with these systems include the limited repairability, which is regulated by the ratio of microcapsules incorporated into the system [6]. Furthermore, as higher ratios of microcapsules are added to the polymeric network, there is a compromise in the bulk mechanical properties due to the lack of chemical bonding between the microcapsule shells and the organic matrix. Strategies to overcome these limitations include incorporating high toughness compounds into the healing agent systems to enhance the overall mechanical properties of the systems and functionalizing the surface of the microcapsules to establish covalent bonds with the organic matrix. The implementation of advanced technologies to facilitate effective, consistent, and homogenous encapsulation of the healing agents is also instrumental in order to make the synthetic procedures more robust and ensure clinical translation.

**Figure 3:**
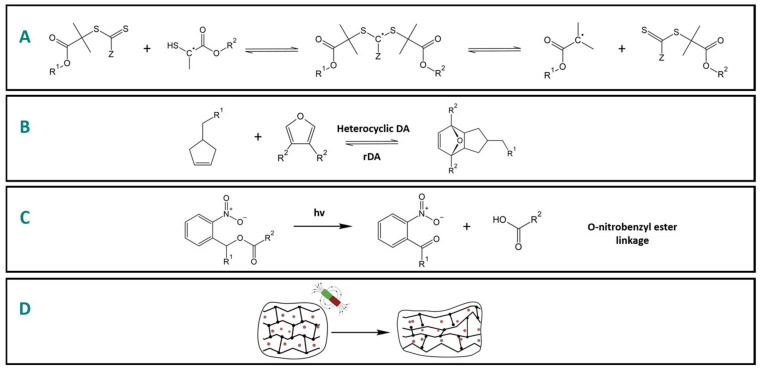
Schematic representation of physical-stimuli-responsive approaches currently employed in dentistry. (**A**) Reversible addition–fragmentation chain transfer reactions employed to mitigate the stress generated during the resin composite polymerization [10]. (**B**) Thermally sensitive hetero Diels–Alder moieties incorporated into a dimethacrylate crosslinker in order to facilitate restorative materials debonding in dental restorations aimed to be replaced [12]. (**C**) O-Nitrobenzyl ester linker incorporated into polyrotaxanes crosslinkers (PRXs) that are cleaved upon exposure to UV light with the goal of facilitating failed dental restoration removal [16]. (**D**) Schematic representation of the magnetic field that has been utilized to generate heat to facilitate network recovery in thermo-responsive shape memory polymers [18].

Intrinsic approaches have been also employed in the Dental field but primarily as a strategy to reduce the stress generated during the light-activated polymerization of dental composites. One of the strategies relies on reversible addition fragmentation chain transfer (RAFT) reactions, which involve the utilization of an addition fragmentation compound capable of cleaving and re-forming covalent bonds through reactive fragments prior to reaching the gel point [10] (Figure 3A). By strategically incorporating these compounds, the network structure of the polymer can undergo controlled fragmentation and subsequent reformation, thereby allowing for stress relaxation and improved mechanical properties in the resulting polymer. Thiourethane-containing networks have also demonstrated significant potential in terms of bonds reprocessing through associative and dissociative reversible reactions [11]. Although the precise underlying reprocessable mechanism in thiourethane-containing dental polymers is not yet fully understood, it likely contributes to accelerated and enhanced stress relaxation [11]. Further research is needed to comprehensively elucidate the dynamic chemical reactions underlying the observed effects and explore the full potential of the incorporation of thiourethane oligomeric additives in dental restorative materials.

#### 2.1.2. Temperature

Thermo-responsive polymers exhibit a clinical solution temperature at which the polymeric network undergoes a phase change, triggered by disruptions in intra- and intermolecular interactions, resulting in microstructural rearrangements [1]. The Tg, which denotes the temperature range above which an amorphous polymer transitions from a hard or glassy state to a rubbery/viscous state, along with the free volume (the unoccupied volume within a polymeric network), plays a crucial role in this dynamic [2]. The characteristics and the ratio of the responsive molecular moieties incorporated into polymeric networks also play a crucial role in determining the responsivity of the systems [2]. The incorporation of both hydrophilic and hydrophobic moieties within the same network has demonstrated enhanced responsivity, which is likely attributed to changes in entropy that govern the rearrangement of macromolecular segments [2]. Furthermore, the spatial configuration of the structural components exhibiting rebonding or conformational changes, as well as the specific mechanism or type of attachment employed, are critical considerations in the development of thermo-responsive polymers. These factors impact shape matching, mechanical interlocking, and long-range interactions, ultimately influencing the overall performance of the materials [2].

The primary obstacle encountered in the development of thermo-responsive dental composites lies in the inherently high Tg exhibited by their polymeric networks, which is essential for maintaining the mechanical and physical properties required in the challenging oral environment. Consequently, in order to facilitate conformational rearrangements, it becomes necessary to elevate the temperature of the material to ranges that are not physiologically compatible. This requirement poses a significant challenge as it hinders the practical application of thermo-responsive dental composites. A promising strategy to overcome this challenge involves grafting stimuli-responsive segments with low Tg onto the polymer backbone. This approach imparts enhanced mobility to the system at lower temperatures, leading to increased free volume, which in turn creates more favorable spatial conditions for conformational rearrangements of the polymeric chains [2]. Another alternative strategy involves incorporating moieties that are responsive to different stimuli and can undergo localized exothermic reactions. This approach has the potential to induce localized and microscaled temperature increases, facilitating the desired structural rearrangements within the material. By exploring this approach, it may be possible to address the temperature limitation issue and enhance the applicability of thermo-responsive dental composites.

Currently, in the field of dentistry, thermally degradable moieties, such as hetero Diels–Alder (HDA) functionalities, have been employed to facilitate the debonding process of failed dental restorations that require replacement [12] (Figure 3B). Heating a dimethacrylate crosslinker containing two thermally sensitive HDA moieties at 80 °C for 3 min leads to bonding cleavage via the retro HDA reaction [12]. This thermal activation results in the breakdown of the crosslinker, thereby aiding in the efficient debonding of the dental restoration and minimizing the undesirable removal of healthy dental tissues [12].

#### 2.1.3. Light

Photoresponsive polymers undergo chemical and physical microstructural changes upon light exposition [13]. These stimuli-responsive systems exhibit remarkable spatial specificity and can respond to a wide range of wavelengths, spanning from ultraviolet (UV) to infrared (IR), which makes them highly versatile [13]. Typically, these stimuli-responsive polymers are categorized as either UV sensitive or visible light sensitive [14]. The design of photoresponsive systems involves a crucial step of selecting appropriate photoreactive functional groups, in addition to considering factors such as glass transition temperature (Tg) and free volume space, as discussed previously [2,13]. In essence, the photoreactive moieties, also known as chromophores, absorb light energy and undergo photoinduced reactions such as isomerization, dimerization, or cleavage [13,15]. These reactions generate a chemical signal, which is then transmitted to the photoactive component of the polymer network. In response, the network undergoes reversible or irreversible conformational changes. The magnitude and nature of the response can be finely tuned by varying the type of chromophore or the specific stimulus parameters, such as light wavelength, intensity, and exposure time [13]. It is instrumental to maintain bonding stability between the chromophore and the polymeric network, as non-covalently bonded species can lead to inconsistent and sluggish responses, ultimately impacting the efficiency of the system [14].

In the field of dentistry, the integration of photodegradable polyrotaxanes crosslinkers (PRXs) into polymeric materials has been explored to facilitate the debonding process of failed dental restorations [16] (Figure 3C). These PRXs are considered supramolecular-interlocked polymers, comprising α-cyclodextrin threaded onto a linear poly(ethylene glycol) chain and terminated with bulky stopper molecules. When exposed to ultraviolet (UV) light, the o-nitrobenzyl ester linkers within the PRXs undergo cleavage, resulting in the release of the bulky stopper molecules. This cleavage event leads to a significant 60% decrease in tensile bond strength, which facilitates the debonding of restorative materials and makes the restoration removal procedures much less invasive [16].

#### 2.1.4. Magnetic/Electric Field

Magnetic-field-responsive polymers exhibit the ability to adaptively modify their physical properties when subjected to an external magnetic gradient [17]. This magnetic responsiveness is primarily achieved through the incorporation of micro- or nano-sized magnetic filler particles into the polymer matrix [18]. The presence of these magnetic particles enables conformational changes through atomic or subatomic responses, resulting in the creation of a magnetic dipole moment as a result of electron motion [18]. These magnetic moments arise from the spin of electrons around their own axes or the orbital movement of electrons around the nucleus of atoms, all occurring in the same direction [18].

The design parameters for magnetic-field-responsive polymeric systems depend on the specific clinical application. In the context of restorative dentistry, magnetically actuated thermoresponsive polymers with controlled shape shifting represent a promising strategy within this category of responsive systems [18] (Figure 3D). In such systems, the thermal energy generated by the repeated alignment of magnetic spins and subsequent relaxations is harnessed to induce phase transitions in shape memory polymers [18]. A notable advantage of this approach is the ability to remotely induce localized temperature changes without direct contact [18]. To achieve optimal performance, it is recommended that one create a single domain of magnetic nanoparticles possessing intrinsic superparamagnetic properties. This ensures no dipolar attraction in the absence of external magnetic flux, which leads to improved colloidal stability and facilitates their uniform dispersion within the organic matrix [18]. In these systems, the magnetic fillers should be loaded at high ratios, be homogeneously distributed, and exhibit a high specific absorption rate to optimize the heating source [18]. Conversely, the polymeric matrix should possess effective heat dissipation control capabilities [18].

Similar to other field-responsive systems, polymers sensitive to an electric field exhibit changes in size or shape in response to the applied field [19,20]. These electro-responsive systems can be broadly categorized into two types: ionic or dielectric electro-responsive materials. Ionic systems rely on the mobility of free ions under the influence of the electric field, leading to changes in the local concentration of ions and the subsequent creation of an electric field [19,20]. Generally, these systems operate in wet conditions, require low actuation voltages, and exhibit low deformation rates during slow response [19,20]. On the other hand, dielectric systems operate based on the electrostatic or Coulombic forces generated between electrodes to induce the formation of an electric field [19,20]. These systems are designed to function in dry conditions, require high activation fields, and demonstrate high deformation rates during fast response [19,20].

The clinical translation of electro-responsive polymers to dental applications is undoubtedly the most challenging. Firstly, the complexity of the oral environment can affect the stability and performance of electro-responsive materials, which makes it challenging to preserve their functionality over extended periods. Another challenge lies in the design and integration of the necessary electrical components and power sources into the dental restorations. Developing efficient and compact electrical systems that can be safely integrated into dental restorations without compromising their aesthetics and functionality represents a significant engineering challenge. Moreover, the long-term stability and reliability of electro-responsive polymers into the oral cavity need to be carefully addressed. These materials need to resist degradation to ensure they can withstand the exposure to acidic and enzymatic degradation. Lastly, the presence of metallic restorations, such as dental amalgams and metal copings, can interfere with the electrical properties and responsivity of these systems. The conductivity of metallic materials can disrupt or block electrical fields due to shielding effects, which makes it challenging to achieve effective electrical stimulation.

### 2.2. Chemical-Stimuli-Responsive Polymers

Chemically responsive polymers exhibit their responsiveness by undergoing changes in molecular interactions between polymer chains or between solvent molecules and the polymeric chains. These changes are triggered by oscillations in pH, the presence of specific solvents, or alterations in the oxidation state of redox groups [1]. Chemical stimuli induce changes in the intermolecular forces, such as hydrogen bonding, electrostatic interactions, and hydrophobic interactions, which plays a pivotal role in governing the behavior of these polymers. These alterations in molecular interactions lead to conformational changes, swelling or shrinking of the polymer, or even the release or uptake of molecules from the surrounding environment.

#### 2.2.1. pH

pH-responsive polymers possess ionizable acidic or basic moieties, which enable them to exhibit responsiveness to oscillation in pH through changes in their structure and property [21]. These polymers are classified as polyelectrolytes with weak acidic or basic groups that can accept or release protons in response to changes in the surrounding pH. Consequently, these pH-responsive polymers undergo alterations in surface activity, chain conformation, and solubility [4,21]. Two primary groups of pH-responsive polymers can be distinguished based on the presence of ionizable pendants or acid-/base-labile linkages within their chemical structure [4]. Polymers with ionizable pendants respond to pH variations by accepting or donating protons, resulting in changes in the net charges on the polymer chains and influencing their hydrodynamic volume and conformation [4]. Typically, an increase in the net charge leads to electrostatic repulsion, causing the polymeric network to transition from a collapsed to an expanded configuration and rendering it more hydrophilic [4]. However, in most cases, this phenomenon is reversed as the net charge decreases. On the other hand, polymers with acid-/base-labile linkages undergo reversible or irreversible cleavages of specific segments of their backbones in response to pH variations [4]. The pH transition in both systems is governed by the pKa (for acidic compounds) or pKb (for basic compounds) of the moieties incorporated into the polymer structure. Consequently, careful consideration of the pKa or pKb values is essential to ensure that the polymer exhibits responsiveness within the desired pH range, and the resulting conformational changes triggered by electrostatic interactions align with the intended therapeutic goal [4]. For instance, polyacids exhibit a collapsed polymeric structure when the environmental pH is lower than their pKa, while an expanded polymeric structure is observed when the environmental pH exceeds their pKa [4]. Conversely, polybases demonstrate an inverse response, displaying an expanded structure at lower environmental pH values compared to their pKb, and a collapsed configuration as the environmental pH surpasses their pKb [4]. pH-responsive acidic polymers incorporate weak acidic or basic pendant groups that accept protons at low pH and deprotonate at neutral and high pH values. This characteristic allows for the modulation of hydrophilicity/hydrophobicity, precipitation/solubilization, and swelling/deswelling behaviors [21]. pH-responsive basic polymers, on the other hand, contain weak basic groups that undergo ionization/deionization transitions in the pH range from 7 to 11. They accept protons at neutral or lower pH values and release them as the environmental pH becomes increasingly alkaline [21]. A wide range of polymer architectures can be utilized for designing pH-responsive polymers, with the selection guided by the specific application requirements [21]. Among these architectures, brush, comb, dendrimer, and (hyper)branched polymers appear to be particularly compatible with thermoset dental polymers, offering potential benefits in dental applications [21].

**Figure 4:**
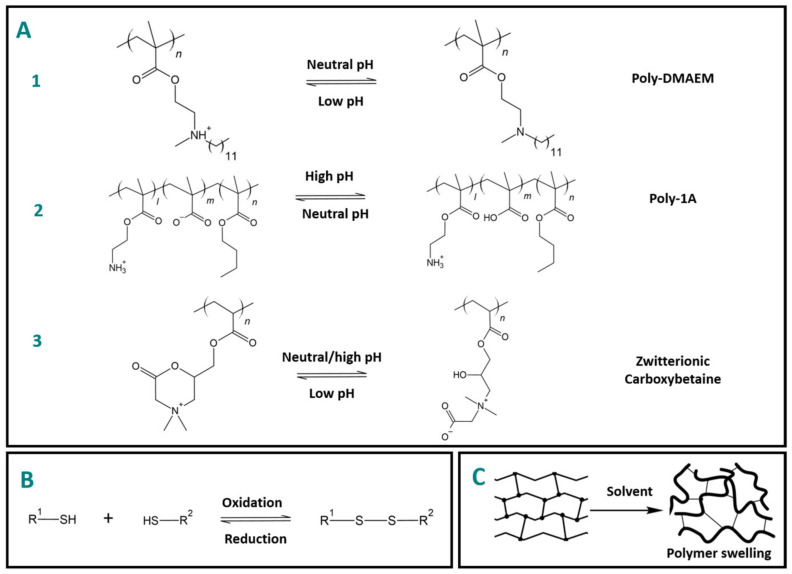
Schematic representation of chemical-stimuli-responsive approaches currently employed in dentistry. (**A1**) Dodecylmethylaminoethyl methacrylate (DMAEM) undergoes protonation, resulting in a positive charge, as the pH decreases, thereby eliciting antimicrobial effects [22]. (**A2**) Poly-1A is a compound that exhibits both positive and negative charges. As the pH decreases and becomes more acidic, the negative charges on Poly-1A are neutralized, resulting in a net positive charge, which endows the material with antimicrobial properties [23]. (**A3**) Carboxybetaine methacrylate zwitterionic compound that transitions from linear to a ring conformation change, which enables it to act as antifouling and antimicrobial agent as the pH oscillates from neutral to acidic [24,25,26]. (**B**) Potential disulfide–thiol redox in thiourethane-containing networks [34]. (**C**) Schematic representation of the swelling mechanism in polymeric networks’ response to solvents.

In dentistry, a pH-sensitive strategy has been used to design antimicrobial and antifouling restorative materials. The approach relies on using the pH oscillations presented in the formation of bacterial biofilm to trigger conformation changes that result in the desired antimicrobial or antifouling effect. In the oral microbiome, caries-causing bacteria are acidogenic, thus lowering the local pH. Integration of dodecylmethylaminoethyl methacrylate (DMAEM), a monomer containing a responsive tertiary amine group, into dental resins has been investigated due to its antimicrobial effect against Streptococcus mutans [22] (Figure 4(A1)). Inclusion of DMAEM in Clinpro™ Sealant at 2.5–5% resulted in a significant reduction of bacterial viability and biofilm metabolic activity [22]. Another pH-responsive switching polymer, Poly-1A, consisting of both cationic (NH_3_^+^) and anionic groups (COO^−^) in equal amounts, has also proved to be capable of transitioning from a neutral state (pH 7.4) to a positively charged state as the pH becomes acidic due to the formation of bacterial biofilm [23]. Protonation of the carboxyl groups of the polymer, results in a net positive charge, thereby providing antimicrobial activity [23] (Figure 4(A2)).

Switchable antimicrobial and antifouling carboxybetaine-based systems are also a compelling approach that has been integrated into dental composite formulations. Carboxybetaine methacrylate (CBMA) possesses a zwitterionic nature due to the presence of both positive and negative charges on the betaine moiety [24,25,26] (Figure 4(A3)). This characteristic imparts switchable behavior to the polymer, allowing it to exhibit different properties in response to environmental stimuli [24,25,26]. For example, at neutral pH and physiological conditions, the carboxybetaine-based polymer surface is highly hydrated and exhibits a “brush-like” conformation, which prevents bacterial adhesion and biofilm formation [24,25,26]. This antifouling property helps to inhibit bacterial colonization on the surface. However, as the pH becomes more acidic, the carboxybetaine-based polymer undergoes a conformational change [24,25,26]. This change exposes the positive charges on the polymer surface, leading to electrostatic interactions with the negatively charged bacterial membranes. The interaction disrupts the membrane integrity, causing cell lysis and resulting in antimicrobial activity against the adhered bacteria [24,25,26].

The main additional challenge in incorporating pH-responsive functional moieties into dental composites is to ensure the inertness of the systems upon subjection to the typical pH oscillations resulting from food and beverage intake. Moreover, ensuring physical availability of the pH-responsive moieties on the polymer surface to effectively interact with bacterial biofilms, rather than being buried within the polymeric network, further compounds the complexity of this task.

#### 2.2.2. Redox

Redox-sensitive polymers are materials responsive to changes in oxidation state induced by the environment in sensitive groups [1]. The two main approaches to design these responsive polymers are to add the oxidation or reducing sensitive groups on the backbone chain of the polymer or as crosslinking agents [27]. While oxidant-responsive polymers are characterized by high free radical reactivity and oxidation potential, reductant-responsive polymers exhibit low free radical reactivity and high reduction potential [28,29,30]. In general, redox-sensitive polymers are highly modifiable and selective and the rearrangements in chemical structure induced by the presence of redox-modulating agents lead to changes in properties, such as hydrophilicity/hydrophobicity, or cleavages of chemical bonds [29]. Given the distinctive oxidative stress found in tumor microenvironments, redox-responsive polymers have been widely used to design membranes to sequester and locally deliver therapeutic agents [29,30]. It is also a promising strategy to enable polymers with self-healing capability by host–guest supramolecular interactions responsive to changes in oxidation state [31]. In these systems, redox stimulus is used to induce sol–gel phase transition in the polymeric network and, ultimately, create sufficient system mobility to favor bond reformation [31].

In the field of dentistry, the incorporation of redox-responsive functionalities into polymeric networks remains relatively unexplored. One of the primary challenges associated with this approach is the influence of saliva on electron transfer processes [32]. Electrolytes present in saliva can serve as charge carriers, facilitating electron transfer. However, enzymes such as peroxidases and reductases can inadvertently participate in electron transfer by either accepting or donating electrons [32]. Additionally, proteins and mucus can impede electron transfer by forming a passivating layer on surfaces [32]. Furthermore, the characteristic fluctuations in pH found in the oral environment can modulate the rate and direction of electron transfer reactions due to alterations in the ionization state and the redox properties of chemical species [33].

Nevertheless, within the context of dynamic reactions occurring in thiourethane-containing reprocessable dental networks, one potential reaction mechanism of interest is the disulfide–thiol exchange reaction, which can proceed through a series of redox reactions. In this mechanism, the disulfide bond is reduced, leading to the formation of two thiol groups, or alternatively, two thiol groups can be oxidized to form a disulfide bond (Figure 4B). In the presence of a reducing agent, such as a thiol compound, the reducing agent donates electrons, causing the disulfide bond to break and resulting in the generation of two thiols [34]. Conversely, in the presence of an oxidizing agent, such as a disulfide compound, the oxidizing agent accepts electrons from the thiol groups, leading to the formation of a disulfide bond [34]. Another intriguing approach is based on poly(glycidyl methacrylate) (PGMA) particles functionalized with disulfide groups, which serve as crosslinking agents [35]. This approach was utilized for the development of redox-active particles capable of electrochemical energy storage through a reversible two-electron reduction of the disulfide bond [35]. While this strategy has primarily been investigated for its potential in energy storage applications, its incorporation into multi-responsive systems that combine redox and electrical-field stimuli holds promising prospects.

#### 2.2.3. Solvent

Solvent-responsive polymers are a class of materials that exhibit specific interactions with solvent molecules, leading to conformational changes in their macromolecular polymer chains [1]. To achieve precise control over the polymer structure, it is crucial to design systems where there is chemical compatibility between the polymeric structure and the solvent molecules [36]. Typically, when polymeric surfaces are exposed to solvents, there is adsorption of the solvent molecules on the surface and subsequent permeation through the network and retention, which results in swelling and, eventually, in the dissolution of the polymer [36]. However, this dissolution process is undesirable as it compromises the mechanical performance and stability of the materials [36]. The key challenge in designing solvent-responsive polymers lies in imparting the surface with the capability of interacting with solvent molecules, enabling a controlled response based on programmed reorientation but preserving network integrity. While some degree of swelling is necessary to confer flexibility to the macromolecular polymer chain [1], it must be carefully controlled to prevent polymer dissolution. Strategies for designing solvent-responsive polymers include the incorporation of zwitterionic groups, microphase-separated domains, and grafted polymer brushes on the polymer surfaces [36].

An interesting approach involves utilizing solvents, such as moisture, as stimuli to induce the recovery process in shape memory polymers [37] (Figure 4C). In contrast to thermally activated shape memory polymers that regain their original undeformed state upon heating above the glass transition temperature (Tg) or melting temperature (Tm), solvent-driven responsive polymers exhibit a recovery mechanism wherein the polymeric networks revert to an intermediate shape that can subsequently reassume the original undeformed state through deswelling processes [37]. This approach holds particular interest for dental adhesives, given their continuous exposure to water in the dentinal tubules.

### 2.3. Biological-Stimuli-Responsive Polymers

Polymers responsive to biological stimuli operate based on molecular functional changes triggered by the presence of specific biomolecules such as glucose, enzymes, or cytokines [1]. Unlike many other stimuli-responsive systems, this category of polymers does not require an external stimulus to initiate the desired responses, as the target biomolecules are naturally and readily available within the biological microenvironment [38]. However, the availability and concentration of biomolecules vary greatly among individuals, posing a significant challenge for these systems to reach consistency. Achieving precision-controlled responses necessitates dynamic adaptation to the local environment, adding further complexity to the design and implementation of these responsive polymers [39].

#### 2.3.1. Glucose

Glucose-responsive polymers are compounds that exhibit the ability to detect upregulated levels of glucose and undergo conformational changes in their polymeric chain networks in response [2,40]. These systems can operate through three main mechanisms: glucose oxidation by glucose oxidase (GOx), glucose-binding protein interactions, or reversible covalent bond formation involving boronic acids [2,40]. It should be noted that systems based on glucose oxidation mediated by GOx are not directly responsive to the presence of glucose itself but rather to the local pH changes resulting from the by-products generated during the oxidation reaction [40]. The glucose-binding protein approach involves incorporating specific proteins into the polymeric networks that selectively bind to free glucose molecules. This approach can be employed to release initially conjugated therapeutic agents or induce gel–sol transitions within the polymeric network [2]. Similarly, the boron-based strategy, which operates through a reversible mechanism, allows for the design of switchable systems that can be turned on and off [2,40]. Furthermore, the water solubility of boronic acids is dependent on the concentration of compatible diols (1,2- and 1,3-diols) and the pH of the local environment [2]. In general, these strategies have been utilized to engineer polymeric membranes containing glucose-sensing moieties, which initially possess a positive charge but undergo a reversible transition to a negative charge as the glucose concentration increases. Consequently, such systems exhibit reversible transitions between hydrophilic and hydrophobic states [40].

**Figure 5:**
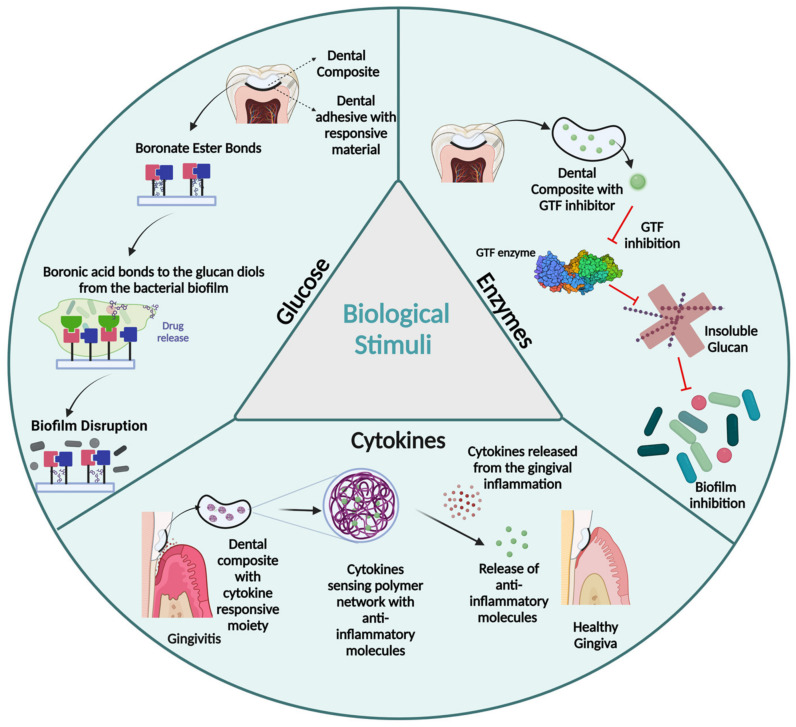
Illustration of biological stimuli approaches that have been employed in the Dental field, as well as the ones with promising potential for future utilization. These approaches encompass a diverse range of innovative techniques and mechanisms that aim to harness the distinct stimuli present in the oral environment for the purpose of enhancing dental treatments.

The development of glucose-responsive polymers specifically tailored for dental applications is an area of ongoing research. While glucose-responsive polymers have been extensively investigated in the field of biomedical engineering and drug delivery systems, their application in dental materials remains relatively limited. However, the concept of incorporating glucose-responsive moieties into dental materials holds promising potential, particularly in the case of dental adhesives. The ability to create adhesive interfaces capable of sensing and responding to glucose levels in the oral cavity or even the diol groups present in glucans within bacterial biofilms could be utilized to prevent or disrupt biofilm formation, ultimately leading to the prevention of bacterial recolonization on dental tissues. Boronate ester compounds also offer a potential approach for incorporating antimicrobial properties into dental resin composites. These compounds have the ability to interact with diol groups found in the glucans present in bacterial biofilms. By incorporating boronate ester compounds into dental resin composites, an exchange reaction is undergone, leading to the release of antimicrobial agents. This mechanism allows for the targeted antimicrobial action against the biofilm, reducing the risk of bacterial colonization and promoting the improved longevity of dental restorations (Figure 5).

#### 2.3.2. Enzymes

Enzyme-responsive polymers undergo reversible or irreversible changes in structure or functionality in response to direct interaction with enzymes under mild conditions [38]. They are highly selective and efficient given the specificity of the enzyme–substrate interactions, which can lead to polymer assemblies, conformational reorganization, and gel–sol/sol–gel transition [38]. Typically, these systems show a polymeric substrate mimic portion designed to be recognized by the target enzyme and interact with it, or a polymeric component that directs and controls changes in supramolecular interactions which leads to macroscopic transitions [38,41].

While enzyme-responsive systems have found widespread applications in drug delivery and tissue engineering, their utilization in restorative polymers is still in its early stages. A pioneering and promising approach involves functionalizing enzyme inhibitors and covalently bonding them to the polymeric network, thereby inducing an ordered response. This approach has been explored in the development of antibiofilm dental resin composites by incorporating glucosyltransferase (GTF) inhibitors [42], which suppress the synthesis and secretion of insoluble glucans that are secreted by cariogenic bacteria and are associated with bacterial adherence and biofilm formation [42] (Figure 5).

Another emerging area of research in dental materials is the development of enzyme-responsive dental adhesives. A promising approach involves the incorporation of mesoporous silica nanoparticles (MSN) loaded with octenidine dihydrochloride (OCT) into dental adhesive systems [43]. These formulations have shown efficient prevention of *S. mutans* biofilm formation, a key contributor to dental caries development [43]. The mechanism of action relies on leveraging the susceptibility of ester bonds to enzymatic degradation by salivary esterases, thereby promoting the release of the loaded drug [43]. However, it should be noted that this drug release mechanism is associated with the degradation of the polymeric structure. While the degradation occurs spontaneously due to the exposure of ester functional groups to water and esterases, it compromises the integrity of the polymeric structure. Further investigations are necessary to optimize the balance between drug release and the maintenance of the structural integrity of enzyme-responsive dental adhesives. Efforts are also focused on developing strategies to enhance the stability and longevity of these materials while preserving their responsiveness to enzymatic degradation for controlled drug release and prevention of biofilm formation. Additionally, enzyme-responsive dental polymers have been investigated for their potential in remineralization and repair of dental tissues. By incorporating enzymes or enzyme-like moieties into the polymers, researchers aim to create systems that can facilitate the deposition of mineral ions and promote the regeneration of tooth structures.

#### 2.3.3. Cytokines

Cytokine-responsive polymers are designed to undergo conformational changes in response to variations in the concentration of small proteins secreted by cells for signaling and modulating biological responses [2]. These polymers can be classified into receptor-based, antibody-based, and stimuli-responsive systems [2]. In receptor-based systems, specific receptors capable of selectively binding to cytokines are incorporated into the polymer network, leading to conformational changes or the initiation of signaling cascades [2]. Antibody-based systems involve the integration of antibodies or antibody fragments within the polymer structure, enabling the capture and response to target cytokines through conformational changes, drug release, or modulation of local cytokine activity [2]. Stimuli-responsive systems, on the other hand, respond to microenvironmental changes triggered by cytokines, such as oscillations in temperature, pH, redox potential, and other factors [2].

The field of cytokine-responsive restorative polymers in dentistry is currently at a nascent stage of development, with ongoing research aimed at exploring their potential applications. An intriguing approach in this area involves the incorporation of cytokine-sensing components into restorative materials, thereby endowing them with the ability to detect and respond to specific cytokines associated with pulp or gingival inflammation. This approach holds promise for triggering localized drug delivery or activating regenerative processes in a targeted manner. By incorporating cytokine-sensing elements into restorative materials, it becomes possible to create intelligent systems that can recognize the presence of specific cytokines and initiate an appropriate response. This response could involve the release of therapeutic agents with anti-inflammatory properties to treat pulpitis or gingivitis. Additionally, the activation of regenerative processes, such as enhanced cell proliferation or differentiation, could be facilitated to support tissue repair and regeneration (Figure 5).

### 2.4. Multi-Stimuli Responsive Systems

As previously discussed, the presence of functional groups within or attached to polymer chains determines the responsiveness of polymers intended to interact with and adapt to external stimuli in real time [44]. Polymeric networks that incorporate two or more distinct functional moieties, and thereby exhibit responsiveness to different stimuli, are categorized as dual- or multi-responsive systems [44]. These systems can demonstrate parallel, serial, or causal responsivity [44]. In parallel or orthogonal systems, the response of one functional group is independent of the response of other groups, indicating that these groups operate autonomously [44]. In serial systems, the response of one functional group enhances the responsivity of other groups [44]. In causal systems, the response of one functional group to an external stimulus generates new stimuli that trigger the response of other groups [44]. The incorporation of multiple stimuli is associated with systems that possess enhanced precision, a broader switching window, and altered switching conditions due to the complexity of the polymeric network [44]. Nevertheless, the design of these systems necessitates increased sophistication and intricate synthetic procedures, as the different functional moieties must be chemically compatible and their concentrations in the polymer chains carefully balanced [39]. Furthermore, their responses should be precisely synchronized, and their sensitivity and stability should be maintained in complex biological microenvironments with multiple triggers [39].

In the field of dentistry, the multi-stimuli responsive approach has predominantly been employed in hydrogels and thermoplastic polymeric networks for orthodontic aligners, notably poly(ether urethane). Some of these networks encompass shape memory polymers that demonstrate responsiveness to both thermal and aqueous stimuli [45]. By exploiting their low glass Tg (as low as 50 °C), these materials establish microenvironments conducive to facilitating conformational chain changes at temperatures compatible with physiological conditions. Alternatively, a potentially promising application of multi-responsive systems involves their implementation in a serial manner to elicit micro-localized changes within thermoset polymeric networks. This serial model exhibits the potential to enhance the mobility of microenvironments within the network, consequently facilitating the reforming of bonds.

## 3. Future Perspectives and Conclusions

The incorporation of responsive moieties into thermoset polymeric networks poses a significant challenge due to their inherent rigidity, which imparts desirable mechanical properties but concurrently hinders dynamic responses. In order to overcome this challenge, promising strategies such as the utilization of grafting and surface functionalization as chemical approaches to incorporate stimulus-responsive moieties into the polymer matrix, along with the strategic utilization of multiple stimuli, can be explored. Grafting responsive functional groups onto the polymeric chains offers a potential solution by ensuring stimuli-responsiveness without compromising the mechanical integrity of the network structure. This approach allows for the introduction of dynamic and reversible chemical bonds while preserving the mechanical strength of the material. Additionally, surface functionalization represents a viable alternative that can enhance the mobility and accessibility of functional responsive groups. By selectively modifying the surface of the thermoset polymers, an increased availability of responsive moieties can be achieved, thereby facilitating their responsiveness. Furthermore, the combination of multiple stimuli can be employed to microscopically modify the reaction environment within the polymeric network. By carefully selecting and manipulating these stimuli, spatial restrictions can be overcome, allowing for localized changes and promoting the reforming of bonds or conformational changes at a microscale level. These strategies hold promise in tackling the challenges associated with incorporating responsive moieties into thermoset polymeric networks, offering potential avenues to achieve enhanced dynamic responsiveness while preserving the desirable mechanical properties of the materials.

In addition, the possibility of integrating therapeutic agents with diagnostic functionalities within a single restorative material system may also play a pivotal role in advancing the dental field. By incorporating therapeutic components such as antimicrobial agents, bioactive molecules, or remineralization agents along with diagnostic functionalities, a new generation of restorative materials capable of actively combating oral diseases, promoting tissue regeneration, and detecting oral pathologies early on can emerge. It may represent a paradigm shift in oral healthcare, offering opportunities for precision and patient-tailored therapies.

## Data Availability

Data are available in a publicly accessible repository.

## References

[1] Ganesh V.A., Baji A., Ramakrishna S. (2014). Smart functional polymers—A new route towards creating a sustainable environment. RSC Adv..

[2] Urban M.W. (2019). Stimuli-Responsive Materials: From Molecules to Nature Mimicking Materials Design.

[3] Wei M., Gao Y., Li X., Serpe M.J. (2017). Stimuli-responsive polymers and their applications. Polym. Chem..

[4] Ofridam F., Tarhini M., Lebaz N., Gagnière É., Mangin D., Elaissari A. (2021). pH-sensitive polymers: Classification and some fine potential applications. Polym. Adv. Technol..

[5] Wiggins K.M., Brantley J.N., Bielawski C.W. (2013). Methods for activating and characterizing mechanically responsive polymers. Chem. Soc. Rev..

[6] Blaiszik B., Kramer S., Olugebefola S., Moore J., Sottos N., White S. (2010). Self-healing polymers and composites. Annu. Rev. Mater. Res..

[7] Wu J., Weir M.D., Melo M.A.S., Strassler H.E., Xu H.H. (2016). Effects of water-aging on self-healing dental composite containing microcapsules. J. Dent..

[8] Fugolin A.P., Ferracane J.L., Pfeifer C.S. (2022). Fatigue-Crack Propagation Behavior in Microcapsule-Containing Self-Healing Polymeric Networks. Mater. Des..

[9] Ning K., Loomans B., Yeung C., Li J., Yang F., Leeuwenburgh S. (2021). Influence of microcapsule parameters and initiator concentration on the self-healing capacity of resin-based dental composites. Dent. Mater..

[10] Park H.Y., Kloxin C.J., Fordney M.F., Bowman C.N. (2012). Stress relaxation of trithiocarbonate-dimethacrylate-based dental composites. Dent. Mater..

[11] Fugolin A.P.P., Costa A.R., Lewis S.H., Goulart M., Erhardt M.C., Pfeifer C.S. (2021). Probing stress relaxation behavior in glassy methacrylate networks containing thio-carbamate additives. J. Mater. Chem. B.

[12] Schenzel A.M., Klein C., Rist K., Moszner N., Barner-Kowollik C. (2016). Reversing Adhesion: A Triggered Release Self-Reporting Adhesive. Adv. Sci..

[13] Ahmed F., Nuruzzaman M., Mondal M.I.H., Mondal M.I.H. (2022). 10—Photo-responsive hydrogel-treated fabrics for smart drug delivery systems. Medical Textiles from Natural Resources.

[14] Francis R., Gopalan G.P., Sivadas A., Joy N. (2016). Properties of Stimuli-Responsive Polymers. Biomedical Applications of Polymeric Materials and Composites.

[15] Zhao Y.-L., Stoddart J.F. (2009). Azobenzene-based light-responsive hydrogel system. Langmuir.

[16] Matsunaga S., Tamura A., Fushimi M., Santa H., Arisaka Y., Nikaido T., Tagami J., Yui N. (2020). Light-embrittled dental resin cements containing photodegradable polyrotaxane cross-linkers for attenuating debonding strength. ACS Appl. Polym. Mater..

[17] Filipcsei G., Csetneki I., Szilágyi A., Zrínyi M. (2007). Magnetic field-responsive smart polymer composites. Oligomers-Polymer Composites-Molecular Imprinting.

[18] Thévenot J., Oliveira H., Sandre O., Lecommandoux S. (2013). Magnetic responsive polymer composite materials. Chem. Soc. Rev..

[19] Manouras T., Vamvakaki M. (2017). Field responsive materials: Photo-, electro-, magnetic-and ultrasound-sensitive polymers. Polym. Chem..

[20] Nithin K.S., Sachhidananda S., Shilpa K.N., Sandeep S., Karthik C.S., Raj B.M.J., Siddaramaiah H. (2021). Polymer-based smart composites and/or nanocomposites for optical, optoelectronic, and energy applications: A brief introduction. Polymer-Based Advanced Functional Composites for Optoelectronic and Energy Applications.

[21] Kocak G., Tuncer C., Bütün V. (2017). pH-Responsive polymers. Polym. Chem..

[22] Li H., Huang Y., Zhou X., Zhu C., Han Q., Wang H., Xu H.H., Ren B., Cheng L. (2021). Intelligent pH-responsive dental sealants to prevent long-term microleakage. Dent. Mater..

[23] Bhat R., Godovikova V., Flannagan S.E., Li Y., Seseogullari-Dirihan R., González-Cabezas C., Kuroda K. (2023). Targeting Cariogenic Streptococcus mutans in Oral Biofilms with Charge-Switching Smart Antimicrobial Polymers. ACS Biomater. Sci. Eng..

[24] Cheng G., Li G., Xue H., Chen S., Bryers J.D., Jiang S. (2009). Zwitterionic carboxybetaine polymer surfaces and their resistance to long-term biofilm formation. Biomaterials.

[25] Jin J., Kim J.-Y., Choi W., Lee M.-J., Seo J.-Y., Yu J., Kwon J.-S., Hong J., Choi S.-H. (2020). Incorporation of carboxybetaine methacrylate into poly (methyl methacrylate) to prevent multi-species biofilm formation. J. Ind. Eng. Chem..

[26] Kim D., Lee M.J., Kim J.Y., Lee D., Kwon J.S., Choi S.H. (2019). Incorporation of zwitterionic materials into light-curable fluoride varnish for biofilm inhibition and caries prevention. Sci. Rep..

[27] Rao N.V., Ko H., Lee J., Park J.H. (2018). Recent progress and advances in stimuli-responsive polymers for cancer therapy. Front. Bioeng. Biotechnol..

[28] Lallana E., Tirelli N. (2013). Oxidation-responsive polymers: Which groups to use, how to make them, what to expect from them (biomedical applications). Macromol. Chem. Phys..

[29] Liu J., Jia B., Li Z., Li W. (2023). Reactive oxygen species-responsive polymer drug delivery systems. Front. Bioeng. Biotechnol..

[30] Chen M., Li J.-W., Zhang W.-J., Hong C.-Y., Pan C.-Y. (2019). pH-and reductant-responsive polymeric vesicles with robust membrane-cross-linked structures: In situ cross-linking in polymerization-induced self-assembly. Macromolecules.

[31] Nakahata M., Takashima Y., Yamaguchi H., Harada A. (2011). Redox-responsive self-healing materials formed from host–guest polymers. Nat. Commun..

[32] Humphrey S.P., Williamson R.T. (2001). A review of saliva: Normal composition, flow, and function. J. Prosthet. Dent..

[33] Hollmann B., Perkins M., Chauhan V.M., Aylott J.W., Hardie K.R. (2021). Fluorescent nanosensors reveal dynamic pH gradients during biofilm formation. NPJ Biofilms Microbiomes.

[34] Nagy P. (2013). Kinetics and mechanisms of thiol–disulfide exchange covering direct substitution and thiol oxidation-mediated pathways. Antioxid. Redox Signal..

[35] Grocke G.L., Zhang H., Kopfinger S.S., Patel S.N., Rowan S.J. (2021). Synthesis and Characterization of Redox-Responsive Disulfide Cross-Linked Polymer Particles for Energy Storage Applications. ACS Macro Lett..

[36] Cimatu K.L., Ambagaspitiya T.D., Premadasa U.I., Adhikari N.M., Kruse A., Robertson E., Guan S., Rong L., Advincula R., Bythell B.J. (2022). Polymer-solvent interaction and conformational changes at a molecular level: Implication to solvent-assisted deformation and aggregation at the polymer surface. J. Colloid Interface Sci..

[37] Basak S., Bandyopadhyay A. (2021). Solvent Responsive Shape Memory Polymers-Evolution, Current Status, and Future Outlook. Macromol. Chem. Phys..

[38] Asha A.B., Srinivas S., Hao X., Narain R., Aguilar M.R., Román J.S. (2019). Chapter 5—Enzyme-Responsive Polymers: Classifications, Properties, Synthesis Strategies, and Applications. Smart Polymers and Their Applications.

[39] Fu X., Hosta-Rigau L., Chandrawati R., Cui J. (2018). Multi-stimuli-responsive polymer particles, films, and hydrogels for drug delivery. Chem.

[40] Wang J., Yu J., Zhang Y., Zhang X., Kahkoska A.R., Chen G., Wang Z., Sun W., Cai L., Chen Z. (2019). Charge-switchable polymeric complex for glucose-responsive insulin delivery in mice and pigs. Sci. Adv..

[41] Heinemann M., Sauer U. (2010). Systems biology of microbial metabolism. Curr. Opin. Microbiol..

[42] Scaffa P.M.C., Kendall A., Icimoto M.Y., Fugolin A.P.P., Logan M.G., DeVito-Moraes A.G., Lewis S.H., Zhang H., Wu H., Pfeifer C.S. (2023). The potential use of glycosyl-transferase inhibitors for targeted reduction of S. mutans biofilms in dental materials. Sci. Rep..

[43] Stewart C.A., Hong J.H., Hatton B.D., Finer Y. (2018). Responsive antimicrobial dental adhesive based on drug-silica co-assembled particles. Acta Biomater..

[44] Schattling P., Jochum D.F., Theato P. (2014). Multi-stimuli responsive polymers—The all-in-one talents. Polym. Chem..

[45] Schönfeld D., Koss S., Vohl N., Friess F., Drescher D., Pretsch T. (2023). Dual Stimuli-Responsive Orthodontic Aligners: An in Vitro Study. Materials.

